# The oldest anchor for lunar crater chronology constrained by the new age of 4.25 Ga of the South Pole-Aitken basin

**DOI:** 10.1093/nsr/nwaf164

**Published:** 2025-04-25

**Authors:** Yi-Gang Xu, Jingyou Chen

**Affiliations:** State Key Laboratory of Deep Earth Processes and Resources, Guangzhou Institute of Geochemistry, Chinese Academy of Sciences, China; State Key Laboratory of Deep Earth Processes and Resources, Guangzhou Institute of Geochemistry, Chinese Academy of Sciences, China

The South Pole-Aitken (SPA) basin, with a diameter of ∼2500 km and a depth exceeding 8 km [1], is the largest confirmed impact architecture on the lunar surface. Stratigraphic relationships further suggest it is the Moon's oldest impact basin (undisputed), rendering it a critical benchmark for understanding early lunar evolution [2]. Extensive studies from the Apollo era to the present have revealed the SPA basin's multifaceted significance in addressing fundamental questions in lunar science [3], including determination of early lunar impact flux, e.g., lunar heavy bombardment (LHB) [4], testing of the lunar magma ocean (LMO) hypothesis [5], reconstruction of thermal evolution [6] and understanding lunar dichotomy [7,8]. For these reasons, determining the age of SPA has been identified by the planetary science community as one of the highest lunar science priorities [9].

Before the return of the Chang'e-6 sample, the SPA basin age was mainly determined by lunar cratering chronology, or inferred from radioisotopic ages of lunar samples whose provenances are only loosely constrained. For instance, based on the disconcordant features of zircon U-Pb ages in Apollo 12, 14 and 17, a high-precision zircon age of ∼4.33 Ga was ascribed to possibly representing the SPA impact basin event [10]. The proposed ∼4.33 Ga for the SPA basin age is consistent with the Pb-Pb dating of one lunar meteorite, Northwest Africa 2996, which was suggested to be possibly launched from the SPA region [11]. Ar-Ar analyses of putative SPA basin-origin ejecta suggest a slightly younger age of 4.29 Ga [12] or 4.25 Ga [13] for the SPA basin formation. On the other hand, according to different crater size-frequency distributions, the model age of the SPA basin was estimated to be 4.26 Ga [14] to 4.31 Ga [15]. Apparently, current estimates for the SPA basin's age range from ∼4.25 Ga to ∼4.33 Ga. The large time interval reflects the uncertainty associated with age determination, and thus necessitates confirmation by analyses on samples with known provenance.

The most direct and reliable approach for determining the age of the SPA basin is to analyze a sample within the basin that formed as a result of the SPA impact event or that has an age that was reset during the SPA impact event [9]. The ∼1935 g Chang'e-6 lunar soils sampled from the region of the SPA basin provided an unprecedented opportunity to solve the issue. Obtaining a robust age of the SPA relies on (i) the selection of samples that truly represent materials generated during SPA impact; (ii) identification of appropriate dating materials; (iii) state-of-the-art dating techniques with high space resolution and high analytical precision.

Su *et al.* [16] did an excellent job that fulfilled all these three aspects. Previous studies have led to the consensus that the noritic lithologies represent samples from a melt sheet created during the impact event or from a melted mixture of mantle and lower crustal materials [17]. Su *et al.* [16] identified 20 clasts of noritic composition from allocated Chang'e-6 soil, and many of them contain Zr-bearing materials that are suitable for dating. They found high Ni/Co ratios of FeNi alloy in these fragments and characteristic petrologic features, thereby distinguishing them from differentiated materials from the LMO. They dated the SPA impact to 4.25 Ga based on five norite fragments.

**Figure 1.**
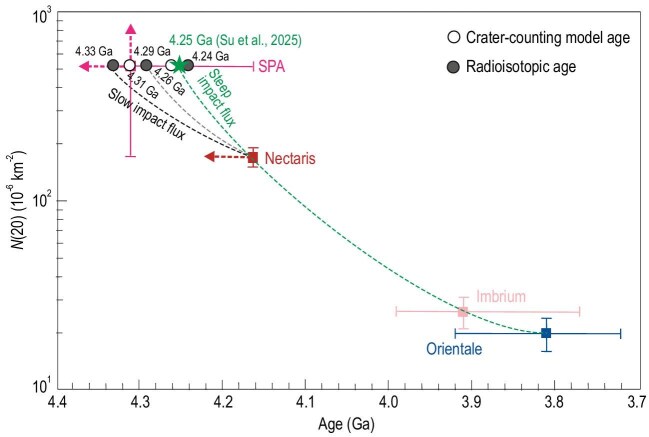
Comparison of early impact flux based on different proposed ages of the SPA basin. A 4.25 Ga SPA basin age indicates that the early impact flux decreased faster and the LHB likely initiated later than those of the previously-proposed 4.3 Ga. Use of *N*(20) and age ranges are from references [2,15]. The previous ages of the SPA basin are from sample analyses [10–13] and lunar crater chronology [14,15].

The newly determined age of 4.25 Ga for the SPA basin revises our understanding of the Moon's earliest impact history. This age provides the oldest anchor point for the lunar cratering chronological curve (Fig. 1). This updated SPA age of 4.25 Ga is slightly younger than the previous model age of 4.3 Ga, suggesting that early lunar impact flux decreased faster and the LHB likely initiated later than previously thought. It also precluded the long-standing hypothesis that the SPA triggered the thermal pulse responsible for widespread lunar magmatism at ∼4.35 Ga [18]. Therefore, the finding by Su *et al.* [16] has significant implications for the dynamics of the Moon, asteroid, terrestrial planet and the solar system.

The study of Su *et al.* [16] will certainly stimulate further discussions and investigations in coming years on key questions such as: (i) **Norite origin:** What is the geological context in which Chang'e-6 norites were formed? How do Chang'e-6 norites definitively link to the SPA impact melt sheet? (ii) **Cooling history:** Considering the heating contribution of elevated Th abundance to the SPA basin [17], how long did the SPA melt pool differentiate? Was a prolonged cooling process recorded by the radioactive isotopic chronometer? (iii) **Survival and delivery mechanism:** Due to several later impact basins superimposed within the SPA basin, how did SPA norites survive later basin-forming impacts, and what was their delivery pathway to the Chang'e-6 site? (iv) **Orbital dynamics:** Orbital dynamics studies suggest that the SPA-scale impactor rarely occurred on the Moon after 4.3 Ga, as the migration of giant planets readily occurred shortly after the formation of the Moon [19]. What orbital configurations allowed such a late, massive impact?
